# Capacity building initiatives for the AD/ADRD clinical research workforce

**DOI:** 10.1002/alz70860_104147

**Published:** 2025-12-23

**Authors:** Rosa V. Pirela‐Mavarez, Claudia Alaniz, Omar Oropeza, Silvia Mejia‐Arango, Jesus D Melgarejo, Gladys E. Maestre

**Affiliations:** ^1^ The University of Texas Rio Grande Valley School of Medicine, Harlingen, TX, USA; ^2^ RGV Alzheimer's Resource Center (AD‐RCMAR), Harlingen, TX, USA; ^3^ South Texas Alzheimer's Disease Research Center, Harlingen, TX, USA; ^4^ Institute of Neuroscience at the University of Texas Rio Grande Valley, Harlingen, TX, USA

## Abstract

**Background:**

The investment in clinical research continues to grow. For Alzheimer's Disease and Alzheimer's Disease Related Dementias (AD/ADRD) only, the National Institute of Health investment grew from 986 million in 2016 to 3.7 billion in 2023. Additionally, the number and complexity of clinical trials is rising. In 2024, there were 127 drugs assessed in clinical trials across the 2024 AD drug development pipeline. This requires a research workforce highly trained, with significant intellectual and skill demands. In minority serving institutions, which have unique opportunities to recruit minority groups into clinical research and where the needs of the workforce may be different, capacity building required tailored approaches. Our research group, dedicated to the study of AD/ADRD in Hispanic populations, implemented capacity building activities for the research staff to conduct high‐impact research, advance community change and promote healthy aging in the Rio Grande Valley, an impoverished area located in Texas/Mexico border. Our objective is to identify capacity building activities for research staff and metrics of success of these initiatives.

**Method:**

Listening sessions were carried out to understand barriers, facilitators, beliefs, and preferences of the clinical research staff. Through the lens of social learning and empowerment theory, a community cultural wealth model served to guide our capacity building efforts to intentionally center cultural values within our learning environment and training activities.

**Result:**

Competing needs and lack of understanding of the dynamics of a multicultural environment and the academia were identified as barriers among research staff. A framework for building capacity for clinical research staff was developed. Two themes: learning environment and quality of training were identified. Each theme is linked to several activities for which specific metrics were developed. Activities included study circles, trainings and experiential learning activities aimed at strengthening their capacity to become healthier, more confident, and self‐empowered research staff.

**Conclusion:**

Capacity building initiatives focused on cultural values and empowerment can foster a resilient and adaptable research workforce poised to thrive in the dynamic AD/ADRD research landscape. In addition, a highly trained, multicultural clinical research workforce can facilitate the development of diverse ideas needed to ultimately improve the AD/ADRD research outcomes.

